# The association between serum ferritin and 25-hydroxyvitamin D and metabolic syndrome in Korean women: the Korea National Health and Nutrition Examination Survey 2010–2012

**DOI:** 10.3164/jcbn.16-115

**Published:** 2017-06-02

**Authors:** Hyun Yoon, Nan Young Bae, Mi Young Gi, Bu Yeon Park, Jeong Min Seong

**Affiliations:** 1 Department of Biomedical Laboratory Science, Hanlyo University, Hanlyo University, 94-13, Hallyeodae-gil, Gwangyange-up, Gwangyang-si, Jeollanamdo, 57764, Korea; 2 Department of Biomedical Laboratory Science, Gwangyang Health College, 111, Hallyeodae-gil, Gwangyang-eup, Gwangyang-si, Jeollanam-do, 57764, Korea; 3 Department of Nursing, Christian College of Nursing, 6, Baekseo-ro 70 beon-gil, Nam-gu, Gwangju, 61662, Korea; 4 Department of Hospital Administration, Seonam University, 439, Chunhyang-ro, Namwon-si, Jeollabuk-do, 55724, Korea; 5 Department of Dental Hygiene, College of Health Science, Kangwon National University, 346, Hwangjo-gil, Dogye-eup, Samcheok-si, Gangwon-do, 25913, Korea

**Keywords:** vitamin D, 25-hydroxyvitamin D, ferritin, metabolic syndrome

## Abstract

The present study was conducted to assess the association between serum ferritin and 25-hydroxyvitamin D [25(OH)D] and metabolic syndrome (MetS) in Korean women. The data of a total of 9,256 adults (6,960 women without MetS and 2,296 women with MetS) aged ≥20 years from the Fifth Korean National Health and Nutrition Examination Survey (KNHANES V) (2010–2012) were analyzed. A covariance test adjusted for covariates was performed for serum ferritin levels in relation to vitamin D (vitamin D deficiency, 25(OH)D <10.0 ng/ml; vitamin D insufficiency, 25(OH)D ≥10.0, <20.0 ng/ml; vitamin D sufficiency, 25(OH)D ≥20.0 ng/ml). The key study results were as follows: First, in women without MetS, after adjusting for related variables (smoking, alcohol drinking, regular exercise, current menstruation, hormonal contraceptives, hormone-replacement therapy, SBP, DBP, BMI, WM, TC, TGs, HDL-C, FPG, AST, ALT, and age), vitamin D was positively associated with serum ferritin levels (*p*<0.001). Second, in women with MetS, after adjusting for related variables (except age), vitamin D was positively associated with serum ferritin levels (*p* = 0.041). However, when further adjusted for age, vitamin D was not associated with serum ferritin levels (*p* = 0.293). In conclusion, vitamin D was positively associated with serum ferritin levels in women without MetS but not in women with MetS.

## Introduction

Iron is a vitally important metal to the normal physiological processes of many organisms,^(1)^ and it is essential for many metabolic processes, such as oxygen transport and DNA synthesis.^(2)^ The serum ferritin level reflects iron stores in the body, since ferrous iron combined with apoferritin is stored by ferritin in many organisms.^(3)^ Low serum ferritin levels are associated with diseases such as telogen effluvium, iron deficiency anemia (IDA), and bone mineral density,^(4–6)^ while high serum ferritin levels are associated with cardiovascular disease, insulin resistance, and metabolic syndrome (MetS).^(7–9)^

In the past, the main role of vitamin D was understood to be controlling the calcium levels and bone metabolism by its involvement in calcium and phosphate absorption in the intestines.^(10)^ However, recently, vitamin D has also received attention regarding additional functions concerning its effects on the prevention of diseases, such as telogen effluvium, cardiovascular disease, MetS, and anemia.^(11–14)^

In terms of anemia, some studies have reported that vitamin D was positively associated with ferritin levels.^(15,16)^ However, these results may vary depending on whether the subjects have diseases such as MetS and diabetes mellitus, because ferritin is a marker of iron stores but also an important biomarker of insulin resistance, inflammation, and oxidative stress.^(17)^ Currently, it is unclear whether ferritin can be considered as a marker of iron stores or inflammation in subjects with insulin resistance. Serum ferritin levels are regulated by hepcidin, which plays a role in reducing iron absorption from the intestine.^(18)^ However, some studies reported that in spite the increase of hepcidin, serum ferritin levels were increased in subjects with MetS.^(19,20)^ Therefore, our objective in this work was to assess the association between vitamin D and ferritin levels in Korean women with and without MetS using data from the Fifth Korean National Health and Nutrition Examination Survey (KNHANES V; 2010–2012), which is representative of the population of Korea (https://knhanes.cdc.go.kr/knhanes/index.do).

## Materials and Methods

### Study subjects

This study was performed using data from KNHANES V. KNHANES V were each conducted for 3 years (2010–2012), using a rolling sampling survey that involved a complex, stratified, multistage, probability cluster survey of a representative sample of the non-institutionalized civilian population in South Korea. The survey was composed of three parts: a health interview survey, a health examination survey, and a nutrition survey. Each survey was conducted by specially trained interviewers. The interviewers were not provided with any prior information regarding specific participants before conducting the interviews. Participants provided written informed consent to participate in this survey, and we received the data in anonymized form. In the KNHANES V (2010–2012), 25,534 individuals over age 1 were sampled for the survey. Among them, of the 19,392 subjects who participated in the KNHANES V, we limited the analyses to adults aged ≥20 years. We excluded participants 2,138 subjects whose data were missing for important analytic variables, such as serum ferritin level, 25(OH)D level, various blood chemistry tests, and information about lifestyle. We excluded participants who had cancer (650 subjects) or hepatitis virus B (470 subjects) or hepatitis virus C (38 subjects). In addition, we excluded participants were excluded 6,840 men. Finally, 9,256 subjects were included in the statistical analysis. The KNHANES V study has been conducted according to the principles expressed in the Declaration of Helsinki. (Institutional Review Board No. 2010-02CON-21-C; 2011-02CON-06-C; 2012-01EXP-01-2C). All participants in the survey signed an informed written consent form. Further information can be found in “The KNHANES V Sample”, which is available on the KNHANES website. The data from KNHANES is available on request by email if the applicant logs onto the “Korea National Health and Nutrition Examination Survey” website.

### General characteristics and blood chemistry

 Research subjects were classified by sex (men or women), smoking (non-smoker or ex-smoker or current smoker), alcohol drinking (yes or no), and regular exercise (yes or no). In the smoking category, participants who smoked more than one cigarette a day, those who had previously smoked but do not presently smoke, and those who never smoked were classified into the current smoker, ex-smoker, and non-smoker groups, respectively. Alcohol drinking was indicated as “yes” for participants who had consumed at least one glass of alcohol every month over the last year. Regular exercise was indicated as “yes” for participants who had exercised on a regular basis regardless of indoor or outdoor exercise. (Regular exercises was defined as 30 min at a time and 5 times/w in the case of moderate exercise, such as swimming slowly, doubles tennis, volleyball, badminton, table tennis, and carrying light objects; and for 20 min at a time and 3 times/w in the case of vigorous exercise, such as running, climbing, cycling fast, swimming fast, football, basketball, jump rope, squash, singles tennis, and carrying heavy objects). Women health index were included current menstruation, hormonal contraceptives, and hormone-replacement therapy.

Anthropometric measurements included measurement of body mass index (BMI) and waist measurement (WM), as well as final measurements of systolic blood pressure (SBP) and diastolic blood pressure (DBP). Blood chemistries included measurements of aspartate aminotransferase (AST), alanine aminotransferase (ALT), total cholesterol (TC), high density lipoprotein cholesterol (HDL-C), triglycerides (TGs), fasting plasma glucose (FPG), 25-hydroxyvitamin D [25(OH)D], serum iron (Fe), total iron binding capacity (TIBC), transferrin saturation (TFS), hemoglobin (Hb), and hematocrit (Hct).

### Metabolic syndrome

Metabolic syndrome was defined using the diagnostic criteria of the National Cholesterol Education Program (NCEP) based on common clinical measures including TGs, HDL-C, blood pressure, FBG, and WM. TGs over 150 mg/dl was set as the criteria for elevated TGs. The criteria for reduced HDL-C were HDL-C of less than 50 mg/dl. FBG over 100 mg/dl was set as the criteria for elevated FBG. SBP over 130 mmHg or DBP over 85 mmHg or medication were set as the criteria for elevated blood pressure. The criteria for abdominal obesity were abdominal measurements of over 80 cm, according to the Asia-Pacific criteria.^(21)^ The presence of defined abnormalities in any three of these five measures constitutes a diagnosis of metabolic syndrome.

### Serum 25(OH)D and ferritin assessments

Blood samples were collected through an antecubital vein after 10–12 h of fasting to assess serum levels of biochemical markers. Serum levels of 25(OH)D were measured with a radioimmunoassay (25-hydroxy-vitamin D ^125^I RIA Kit; DiaSorin, Still Water, MN) using a 1470 Wizard Gamma Counter (Perkin Elmer, Turku, Finland). To minimize the analytical variation, serum 25(OH)D levels were analyzed by the same institute, which carried out a quality assurance program through the analysis period. Serum 25(OH)D levels were classified as either vitamin D deficiency [25(OH)D <10.0 ng/ml], vitamin D insufficiency [25(OH)D ≥10.0, <20.0 ng/ml], or vitamin D sufficiency [25(OH)D ≥20.0 ng/ml].^(22)^ Concentrations of serum ferritin were measured using an immunoturbidimetric Assay (IRMA-mat Ferritin; DiaSorin, Still Water, MN) using a 1470 Wizard Gamma Counter (Perkin Elmer, Turku, Finland).

### Data analysis

The collected data were statistically analyzed using SPSS WIN (ver. 18.0). The distributions of the participant characteristics were converted into percentages, and the successive data were presented as averages with standard deviations. The distribution and average difference in clinical characteristics according to vitamin D status were calculated using chi-squared and an analysis of variance (ANOVA). In the case of analysis of covariance test (ANCOVA), the 4 models constructed were: 1) adjusted for smoking, alcohol drinking, regular exercise, current menstruation, hormonal contraceptives, and hormone-replacement therapy; 2) further adjusted for SBP, DBP, BMI, and WM; 3) further adjusted for TC, TG, HDL-C, FBG, AST, and ALT; 4) further adjusted for age. The significance level for all of the statistical data was set as *p*<0.05.

## Results

### Clinical characteristics of research subjects

The clinical characteristics of the research subjects are shown in Table 1. In women without MetS (6,960 subjects), the mean serum 25(OH)D level was 16.37 ± 5.73 ng/ml. According to the classification of vitamin D status, 687 (9.9%), 4,749 (68.2%), and 1,524 (21.9%) subjects were classified as having vitamin D deficiency, insufficiency, and sufficiency, respectively. The mean serum ferritin level was 43.32 ± 36.85 µg/L. In women without MetS (2,296 subjects), the mean serum 25(OH)D level was 17.12 ± 6.11 ng/ml. According to the classification of vitamin D status, 200 (8.7%), 1,476 (64.3%), and 620 (27.0%) subjects were classified as vitamin D deficient, insufficient, and sufficient, respectively. The mean serum ferritin level was 65.99 ± 49.00 µg/L.

### Clinical characteristics of subjects according to vitamin D in women with or without MetS

The clinical characteristics of subjects according to vitamin D status in women with or without MetS are shown in Tables 2 and 3. In women without MetS, variables showing a significant difference in the distribution and the mean value in vitamin D status were age (*p*<0.001), AST (*p*<0.001), ALT (*p*<0.001), Fe (*p*<0.001), TIBC (*p*<0.001), TFS (*p*<0.001), Hb (*p*<0.001), Hct (*p*<0.001), and ferritin (*p*<0.001). In women with MetS, vitamin D status was not associated with age (*p*<0.001), Ferritin (*p* = 0.004), and TIBC (*p* = 0.043). However, AST (*p* = 0.149), ALT (*p* = 0.608), Fe (*p* = 0.533), TFS (*p* = 0.344), Hb (*p* = 0.195), and Hct (*p* = 0.169) were not significant.

### Comparisons of 25(OH)D, ferritin, and anemia indices according to age in women with and without MetS

Comparisons of 25(OH)D, ferritin, and anemia indices according to age are shown in Table 4. In women without MetS, ferritin (*p*<0.001), 25(OH)D (*p*<0.001), Hb (*p* = 0.001), Hct (*p* = 0.002) were increased with age increase. In women with MetS, ferritin (*p*<0.001) and 25(OH)D (*p*<0.001) were increased with age increase. However, Hb (*p* = 0.001) and Hct (*p* = 0.002) were decreased with age increase.

### Comparisons of serum ferritin levels and anemia indices according to vitamin D in women with and without MetS

Comparisons of serum ferritin levels and anemia indices according to vitamin D status are shown in Table 5 and 6. In women without MetS, in terms of serum ferritin levels by vitamin D after adjusting for related variables (smoking, alcohol drinking, regular exercise, current menstruation, hormonal contraceptives, hormone-replacement therapy, SBP, DBP, BMI, WM, TC, TGs, HDL-C, FPG, AST, ALT, and age), serum ferritin levels (Means ± SE) were 37.75 ± 1.31 µg/L (95% CI, 35.19–40.31) for vitamin D deficiency, 43.12 ± 0.49 µg/L (95% CI, 42.16–44.09) for vitamin D insufficiency, and 46.81 ± 0.89 µg/L (95% CI, 45.08–48.55) for vitamin D sufficiency. This shows that vitamin D was positively associated with the serum ferritin levels (*p*<0.001). In women with MetS, after adjusting for related variables (except age), vitamin D was positively associated with the serum ferritin levels (*p* = 0.041). However, when further adjusted for age, vitamin D was not associated with the serum ferritin levels (*p* = 0.293). In terms of anemia indices by vitamin D after adjusting for related variables, vitamin D was significantly associated with Fe (*p* = 0.004), TIBC (*p* = 0.013), Hb (*p*<0.001), and Hct (*p*<0.001) in women without MetS. However, vitamin D was not associated with Fe (*p* = 0.817), TIBC (*p* = 0.494), Hb (*p* = 0.378), and Hct (*p* = 0.430) in women with MetS.

## Discussion

The present study investigated the association between serum ferritin and 25(OH)D levels in Korean women with and without MetS using data from KNHANES V conducted in 2010–2012. There were several key findings of this study after adjusting for variables. Vitamin D was positively associated with serum ferritin levels in women without MetS but not in women with MetS (Table 5).

The prevalence of vitamin D deficiency varies across ethnic groups and countries.^(23)^ The prevalence of vitamin D deficiency or insufficiency (<20.0 ng/dl) in our results (77%) is higher than that in Germany (58%)^(24)^ and similar to that in Scotland (78%).^(25)^ Vitamin D is known to prevent osteoporosis, cardiovascular disease, and insulin resistance.^(10,26,27)^ In addition, vitamin D regulates the hepcidin–ferroportin axis in macrophages, and the increase of vitamin D is known to reduce systemic hepcidin levels and ameliorate anemia.^(28)^ Ferritin, which reflects the iron stores in the blood, is regulated by hepcidin.^(29)^

Research on the association between vitamin D and ferritin is being conducted all over the world. Jeong and colleagues^(15)^ and Andıran and colleagues^(30)^ reported that serum 25(OH)D was positively correlated with serum ferritin levels in Korea and the US, respectively. However, Monlezun and colleagues^(31)^ and Castro and colleagues^(32)^ reported that serum 25(OH)D was not associated with serum ferritin levels in adults from the US and Portugal, respectively. These inconsistent results may be due to the different populations, ethnic groups/countries and the different subjects of the studies (e.g., gender, with or without diseases).

Ferritin is a biomarker of iron stores and is reduced in subjects with anemia.^(3,5,8)^ Some studies have suggested that vitamin D is inversely associated with the incidence of IDA in women. Lee *et al.*^(5)^ reported that the odds ratios for iron deficiency [serum ferritin level <12 µg/L and transferrin saturation <16%] and IDA (Hb <13 g/dl and iron deficiency) in subjects with vitamin D deficiency [25(OH)D <15 ng/ml] were 1.86 (95% CI, 1.07–3.22) and 2.59 (95% CI, 1.11–6.07) after controlling for other risk factors in healthy Korean women. In addition, Suh *et al.*^(33)^ reported that 25(OH)D levels (Means ± SE) were lower in Korean women with IDA (Hb <12 g/dl and serum ferritin level <15 µg/L, 14.43 ± 0.24 ng/ml) than in those with non-IDA (Hb ≥12 g/dl and serum ferritin level ≥15 µg/L, 16.40 ± 0.13 ng/ml) (*p*<0.001). In the present study, in women without MetS, the vitamin D was positively associated with ferritin levels. In addition, the vitamin D was positively associated with Fe, Hb, and Hct levels. These results considered that vitamin D has a positive effect on anemia.

Currently, as far as we know, there is no the research on vitamin D and ferritin in subjects with MetS. In health populations, vitamin D increases the ferritin levels by is suppressed hepcidin. However, in subjects with MetS, despite the increased of hepcidin, serum ferritin levels were increased, and both hepcidin and ferritin levels were increased as increasing of MetS components.^(19,20)^ In the present study, ferritin and 25(OH)D level increased in women with MetS than in women without MetS, but Fe and TFS level decreased in women with MetS than in women without MetS. In women with MetS, when adjusting for related variables (except age), the vitamin D was positively associated with ferritin levels. However, when further adjusting for age, it was not statistically significant. In addition, the vitamin D was not associated with all of the anemia-related variables (e.g., Fe, TIBC, Hb, and Hct). Age is associated with vitamin D and ferritin level and incidence of anemia and MetS.^(34–37)^ In the present study, the incidence of MetS was increased as the increased of age in both women with and without MetS. We found that ferritin, 25(OH)D, Hb, and Hct were increased with increase of age in women without MetS. However, in women with MetS, ferritin and 25(OH)D were increased with increase of age but Hb and Hct were decrease.

In fact, the association of anemia and ferritin is differ among the type of anemia. Ferritin is decreased in subjects with IDA but may be increased in subjects with anemia of chronic disease and inflammation.^(38)^ Vitamin D plays a role in both the prevention of anemia and the improvement of insulin resistance and inflammation.^(39,40)^ In subjects with IDA, vitamin D increase ferritin by down-regulation of hepcidin. However, in subjects with anemia of chronic diseases and inflammation, vitamin D may be decreased ferritin for the improvement of inflammation status.^(17,41)^

The MetS is characterized by insulin resistance,^(42)^ and the subjects with MetS are already status with increased inflammation and reduced bet cell function.^(43,44)^ As described above, ferritin is an indicator of inflammation as well as a marker of iron store. Currently, in the subjects with MetS, whether ferritin is a marker of iron store or a marker of inflammation is still unclear. We considered the possibility that vitamin D may detect the degree of ferritin in the blood and regulate the level of ferritin in women. If less than constant level of ferritin in the blood such as women with IDA or without MetS, vitamin D recognizes the ferritin as a marker of iron store and increases ferritin levels by down-regulation of hepcidin.^(45)^ However, if more than constant level of ferritin in the blood such as women with MetS, vitamin D may recognize the ferritin as a marker of inflammation rather than iron store and may reduce ferritin levels by up-regulation of hepcidin, or it is also a possibility that there will be no change in ferritin level as the up-regulation and down-regulation of hepcidin may occur simultaneously. Martinelli and colleagues suggested that although hepcidin was increased in subjects with MetS, ferritin or Hb also were increased.^(20)^ We found that 25(OH)D was not associated with ferritin as well as Fe, TIBC, Hb, and Hct in women with MetS. These results are similar to study of Luo and colleague.^(46)^ Although the subject is not the MetS, in a study of they conducted on subjects with type 2 diabetes mellitus which is characterized by insulin resistance, vitamin D was not associated with ferritin as well as serum iron, iron saturation, and high sensitivity CRP. In the present study, we could not demonstrate the mechanisms that whether vitamin D plays a role the prevention of IDA or plays a role the improvement of inflammation in women with MetS. However, we were able to determine that vitamin D was not associated with ferritin in Korean women with MetS.

The present study has some limitations. First, season is the most important determinant of serum 25(OH)D levels, but the data of the KNHANES V study (2010–2012) did not specify serum 25(OH)D levels according to season. Therefore, season could not be used as adjustment variable. Second, hepcidin is an important determinant of serum ferritin levels. However, hepcidin were not employed in the KNHANES V study. Third, serum calcium concentrations and the daily intake volume of vitamin D are important determinants of serum 25(OH)D levels, but the KNHANES V study did not measure serum calcium concentrations or daily intake volumes of vitamin D. Therefore, serum calcium concentrations and daily intake volumes of vitamin D could not be used as adjustment variables. Forth, we have not been able to investigate the medication for anemia, diabetes, dyslipidemia, and hypertension. Therefore, the medication for anemia, diabetes, dyslipidemia, and hypertension could not be used as adjustment variables. The serum 25(OH)D levels for each season, along with calcium, and hepcidin, should be included as variables for vitamin D status in future studies. Although the present study has these limitations, this is the first reported study to determine the relationship between ferritin and vitamin D in Korean adults without and with MetS. Therefore, more accurate results might be obtained by performing a cohort study by adding these variables.

In conclusion, the present study investigated the association between serum ferritin and 25(OH)D levels in Korean women with and without MetS using data from the KNHANES V conducted in 2010–2012. Vitamin D was found to increase with serum ferritin levels in women without MetS but not in women with MetS.

## Figures and Tables

**Table 1:** Clinical characteristics of research subjects

*n*(%), Mean ± SD					
Variables		Total (*n* = 9,256)	Non-MetS (*n* = 6,960)	MetS (*n* = 2,296)	*p* value
Age (years)	20–39	2,923 (31.6)	2,751 (39.5)	172 (7.5)	<0.001
	40–59	3,489 (37.7)	2,690 (38.7)	799 (34.8)	
	≥60	2,844 (30.7)	1,519 (21.8)	1,325 (57.7)	
Current smoker		491 (5.3)	383 (5.5)	109 (4.7)	0.042
Alcohol drinker		3,499 (37.8)	2,770 (39.8)	627 (27.3)	<0.001
Regular exerciser		768 (8.3)	551 (7.9)	195 (8.5)	0.377
Current menstruation		1,395 (15.1)	910 (13.1)	485 (21.1)	<0.001
Hormonal contraceptives		791 (8.5)	555 (8.0)	236 (10.3)	0.001
Hormone-replacement therapy		729 (7.9)	657 (9.4)	72 (3.1)	<0.001
BMI (kg/m^2^)		23.41 ± 3.52	22.54 ± 3.14	26.02 ± 3.29	<0.001
WM (cm)		78.58 ± 9.93	75.70 ± 8.78	87.32 ± 7.90	<0.001
SBP (mmHg)		117.87 ± 18.11	113.18 ± 15.71	132.08 ± 17.47	<0.001
DBP (mmHg)		74.16 ± 9.90	72.57 ± 9.20	78.98 ± 10.37	<0.001
AST (mg/dl)		20.20 ± 8.02	19.36 ± 7.51	22.76 ± 8.93	<0.001
ALT(mg/dl)		17.34 ± 11.98	15.82 ± 10.93	21.94 ± 13.72	<0.001
TC (mg/dl)		191.17 ± 36.91	187.93 ± 34.76	200.97 ± 41.26	<0.001
TGs (mg/dl)		114.93 ± 80.50	91.80 ± 49.51	185.03 ± 110.28	<0.001
HDL-C (mg/dl)		55.09 ± 12.84	58.22 ± 12.17	45.63 ± 9.84	<0.001
FPG (mg/dl)		95.70 ± 20.25	91.01 ± 13.80	109.92 ± 28.41	<0.001
Ferritin (µg/L)		48.95 ± 41.39	43.32 ± 36.85	65.99 ± 49.00	<0.001
Fe (µg/dl)		101.19 ± 41.60	102.29 ± 43.54	97.85 ± 34.88	<0.001
TIBC (µg/dl)		321.90 ± 47.56	321.48 ± 48.17	323.18 ± 45.66	0.127
TFS (%)		32.27 ± 13.87	32.73 ± 14.54	30.89 ± 11.52	<0.001
Hb (g/dl)		13.04 ± 1.14	12.95 ± 1.14	13.31 ± 1.11	<0.001
Hct (%)		39.20 ± 2.97	38.99 ± 2.94	39.84 ± 2.97	<0.001
25(OH)D (ng/ml)		16.55 ± 5.84	16.37 ± 5.73	17.12 ± 6.11	<0.001
	<10.0	887 (9.6)	687 (9.9)	200 (8.7)	<0.001
	≥10.0, <20.0	6,225 (67.3)	4,749 (68.2)	1,476 (64.3)	
	≥20.0	2,144 (23.1)	1,524 (21.9)	620 (27.0)	

Non-MetS: non-metabolic syndrome, MetS: metabolic syndrome, BMI: body mass index, WM: waist measurement, SBP: systolic blood pressure, DBP: diastolic blood pressure, AST: aspartate aminotransferase, ALT: alanine aminotransferase, TC: total cholesterol, TGs: triglycerides, HDL-C: high density lipoprotein cholesterol, FPG: fasting plasma glucose, Fe: serum iron, TIBC: total iron binding capacity, TFS: transferrin saturation, Hb: hemoglobin, Hct: hematocrit, 25(OH)D: 25-hydroxyvitamin D.

**Table 2:** Clinical characteristics of subjects according to vitamin D in women without MetS

*n*(%), Mean ± SD, (*n* = 6,960)					
Variables		Serum 25(OH)D levels			*p* value
		Deficiency (<10.0 ng/ml) (*n* = 687)	Insufficiency (≥10.0, <20.0 ng/ml) (*n* = 4,749)	Sufficiency (≥20.0 ng/ml) (*n* = 1,524)	
Age (years)	20–39	353 (51.4)	2,003 (42.2)	395 (25.9)	<0.001
	40–59	237 (34.5)	1,891 (39.8)	562 (36.9)	
	≥60	97 (14.1)	855 (18.0)	567 (37.2)	
Current smoker		45 (6.6)	272 (5.7)	66 (4.3)	0.075
Alcohol drinker		250 (36.4)	1,971 (41.5)	549 (36.0)	<0.001
Regular exercise		31 (4.5)	382 (8.0)	138 (9.1)	0.001
Current menstruation		79 (11.5)	479 (10.1)	99 (6.5)	<0.001
Hormonal contraceptives		65 (9.5)	606 (12.8)	239 (15.7)	<0.001
Hormone-replacement therapy		33 (5.8)	339 (7.1)	183 (12.0)	<0.001
BMI (kg/m^2^)		21.95 ± 3.18	22.56 ± 3.14	22.73 ± 3.08	<0.001
WM (cm)		73.63 ± 8.66	75.63 ± 8.73	76.85 ± 8.78	<0.001
SBP (mmHg)		114.10 ± 15.06	112.36 ± 15.14	116.56 ± 17.19	<0.001
DBP (mmHg)		72.02 ± 8.77	72.41 ± 9.25	73.31 ± 9.20	0.001
AST (mg/dl)		18.25 ± 6.82	19.19 ± 7.89	20.39 ± 5.99	<0.001
ALT(mg/dl)		14.49 ± 11.10	15.80 ± 11.54	16.49 ± 8.63	<0.001
TC (mg/dl)		182.73 ± 34.73	187.25 ± 34.65	192.41 ± 34.64	<0.001
TGs (mg/dl)		91.08 ± 48.14	91.59 ± 51.01	92.80 ± 45.20	0.653
HDL-C (mg/dl)		57.41 ± 12.05	58.37 ± 12.18	58.10 ± 12.21	0.138
FPG (mg/dl)		89.84 ± 11.61	90.89 ± 13.78	91.93 ± 14.72	0.002
Ferritin (µg/L)		33.25 ± 28.82	41.89 ± 36.37	52.33 ± 39.69	<0.001
Fe (µg/dl)		96.04 ± 44.92	102.47 ± 44.60	104.53 ± 39.13	<0.001
TIBC (µg/dl)		326.30 ± 50.59	322.77 ± 48.70	315.29 ± 44.75	<0.001
TFS (%)		30.48 ± 14.66	32.68 ± 14.85	33.88 ± 13.35	<0.001
Hb (g/dl)		12.66 ± 1.26	12.95 ± 1.13	13.06 ± 1.09	<0.001
Hct (%)		38.24 ± 3.14	39.00 ± 2.90	39.29 ± 2.92	<0.001

25(OH)D: 25-hydroxyvitamin D, WM: waist measurement, BMI: body mass index, SBP: systolic blood pressure, DBP: diastolic blood pressure, AST: aspartate aminotransferase, ALT: alanine aminotransferase, TC: total cholesterol, TGs: triglycerides, HDL-C: high density lipoprotein cholesterol, FPG: fasting plasma glucose, Fe: serum iron, TIBC: total iron binding capacity, TFS: transferrin saturation, Hb: hemoglobin, Hct: hematocrit.

**Table 3:** Clinical characteristics of subjects according to vitamin D in women with MetS

*n*(%), Mean ± SD, (*n* = 548)					
Variables		Serum 25(OH)D levels			*p* value
		Deficiency (<10.0 ng/ml) (*n* = 200)	Insufficiency (≥10.0, <20.0 ng/ml) (*n* = 1,476)	Sufficiency (≥20.0 ng/ml) (*n* = 620)	
Age (years)	20–39	25 (12.5)	124 (8.4)	23 (3.7)	<0.001
	40–59	68 (34.0)	552 (37.4)	179 (28.9)	
	≥60	107 (53.5)	800 (54.2)	418 (67.4)	
Current smoker		7 (3.5)	81 (5.5)	21 (3.4)	0.243
Alcohol drinker		43 (21.5)	422 (28.6)	162 (26.1)	0.08
Regular exercise		9 (4.5)	132 (8.9)	54 (8.7)	0.104
Current menstruation		9 (4.5)	55 (3.7)	8 (1.3)	0.007
Hormonal contraceptives		41 (20.5)	305 (20.7)	139 (22.4)	0.68
Hormone-replacement therapy		7 (3.5)	140 (9.5)	89 (14.4)	<0.001
BMI (kg/m^2^)		26.18 ± 3.80	26.18 ± 3.34	25.59 ± 2.97	0.001
WM (cm)		87.32 ± 9.09	87.37 ± 7.88	87.21 ± 7.56	0.914
SBP (mmHg)		131.32 ± 18.37	132.04 ± 17.41	132.43 ±17.34	0.729
DBP (mmHg)		79.51 ± 11.06	79.17 ± 10.21	78.37 ± 10.50	0.205
AST (mg/dl)		21.70 ± 8.74	22.76 ± 9.20	23.12 ± 8.29	0.149
ALT(mg/dl)		21.03 ± 12.68	22.06 ± 14.27	21.95 ± 12.69	0.608
TC (mg/dl)		200.41 ± 40.19	202.01 ± 42.41	198.69 ± 38.72	0.238
TGs (mg/dl)		207.31 ± 109.42	187.75 ± 119.22	171.35 ± 83.71	<0.001
HDL-C (mg/dl)		43.09 ± 8.70	45.81 ± 9.70	46.03 ± 10.40	0.001
FPG (mg/dl)		117.72 ± 32.46	110.45 ± 29.54	108.11 ± 23.87	0.147
Ferritin (µg/L)		59.07 ± 49.78	64.88 ± 48.76	70.91 ± 48.97	0.004
Fe (µg/dl)		95.22 ± 37.32	98.16 ± 36.18	97.99 ± 30.67	0.533
TIBC (µg/dl)		328.39 ± 54.82	323.88 ± 45.68	319.83 ± 42.06	0.043
TFS (%)		29.86 ± 12.35	30.88 ± 11.76	31.23 ± 10.64	0.344
Hb (g/dl)		13.18 ± 1.32	13.31 ± 1.08	13.34 ± 1.10	0.195
Hct (%)		39.47 ± 3.35	39.86 ± 2.87	39.91 ± 3.06	0.169

25(OH)D: 25-hydroxyvitamin D, WM: waist measurement, BMI: body mass index, SBP: systolic blood pressure, DBP: diastolic blood pressure, AST: aspartate aminotransferase, ALT: alanine aminotransferase, TC: total cholesterol, TGs: triglycerides, HDL-C: high density lipoprotein cholesterol, FPG: fasting plasma glucose, Fe: serum iron, TIBC: total iron binding capacity, TFS: transferrin saturation, Hb: hemoglobin, Hct: hematocrit.

**Table 4:** Comparisons of 25(OH)D, ferritin, and anemia indices according to age in women with or without MetS

Mean ± SD, (*n* = 9,256)				
Variables	Age (years)			*p* value
	20–39	40–59	≥60	
Non-MetS (*n* = 6,960)				
Ferritin (µg/L)	31.46 ± 27.18	43.48 ± 37.02	64.52 ± 41.93	<0.001
25(OH)D (ng/ml)	15.20 ± 5.06	16.34 ± 5.51	18.52 ± 6.57	<0.001
Hemoglobin (g/dl)	12.89 ± 1.13	12.97 ± 1.18	13.02 ± 1.07	0.001
Hematocrit (%)	38.86 ± 2.88	39.00 ± 2.97	39.20 ± 3.00	0.002
MetS (*n* = 2,296)				
Ferritin (µg/L)	40.32 ± 36.99	58.93 ± 46.52	73.59 ± 50.07	<0.001
25(OH)D (ng/ml)	14.65 ± 4.97	16.63 ± 5.52	17.73 ± 6.52	<0.001
Hemoglobin (g/dl)	13.41 ± 1.04	13.40 ± 1.15	13.24 ± 1.09	0.002
Hematocrit (%)	40.23 ± 2.76	40.08 ± 2.92	39.65 ± 3.00	0.001

**Table 5:** Comparisons of serum ferritin levels according to vitamin D in women with or without MetS

(*n* = 9,256)				
	Serum ferritin levels (µg/L)			
	Model 1	Model 2	Model 3	Model 4
Non-MetS (*n* = 6,960)				
25(OH)D (ng/ml) <10.0	34.19 ± 1.39	35.39 ± 1.37	36.21 ± 1.34	37.75 ± 1.31
	(31.47–36.90)	(32.69–38.08)	(33.59–38.84)	(35.19–40.31)
≥10.0, <20.0	42.18 ± 0.53	42.49 ± 0.52	42.58 ± 0.51	43.12 ± 0.49
	(41.15–43.21)	(41.47–43.51)	(41.59–43.57)	(42.16–44.09)
≥20.0	51.13 ± 0.93	49.84 ± 0.92	49.19 ± 0.90	46.81 ± 0.89
	(49.31–52.95)	(48.03–51.65)	(47.43–50.95)	(45.08–48.55)
*p* value	<0.001	<0.001	<0.001	<0.001
MetS (*n* = 2,296)				
25(OH)D (ng/ml) <10.0	60.34 ± 3.52	61.17 ± 3.52	61.01 ± 3.42	62.64 ± 3.36
	(53.44–67.24)	(54.27–68.07)	(54.30–67.71)	(56.06–69.22)
≥10.0, <20.0	65.22 ± 1.28	65.36 ± 1.28	65.25 ± 1.24	65.70 ± 1.21
	(62.72–67.73)	(62.86–67.87)	(62.82–67.68)	(63.32–68.08)
≥20.0	69.99 ± 1.98	69.56 ± 1.98	69.88 ± 1.93	68.30 ± 1.89
	(66.12–73.88)	(65.67–73.45)	(66.10–73.65)	(64.58–72.01)
*p* value	0.031	0.072	0.041	0.293

Non-MetS: Non-metabolic syndrome, MetS: Metabolic syndrome, Model 1 [Mean ± SE (95%, CI)], adjusted for smoking, alcohol drinking, regular exercise, current menstruation, hormonal contraceptives, and hormone-replacement therapy; Model 2 [Mean ± SE (95%, CI)], further adjusted for SBP, DBP, BMI, and WM; Model 3 [Mean ± SE (95%, CI)], further adjusted for TC, TG, HDL-C, FBG, AST, and ALT; Model 4 [Mean ± SE (95%, CI)], further adjusted for age.

**Table 6:** Comparisons of anemia indices according to vitamin D in women with or without MetS

(*n* = 9,256)				
	Adjusted Hb (g/dl)*	Adjusted Hct (%)*	Adjusted Fe (µg/dl)*	Adjusted TIBC (µg/dl)*
	[Mean ± SE (95%, CI)]	[Mean ± SE (95%, CI)]	[Mean ± SE (95%, CI)]	[Mean ± SE (95%, CI)]
Non-MetS (*n* = 6,960)				
25(OH)D (ng/ml) <10.0	12.72 ± 0.04	38.38 ± 0.11	97.99 ± 1.66	324.52 ± 1.80
	(12.63–12.80)	(38.17–38.60)	(94.74–101.25)	(320.99–328.05)
≥10.0, <20.0	12.95 ± 0.02	38.99 ± 0.04	101.49 ± 0.63	321.75 ± 0.68
	(12.92–12.99)	(38.91–39.08)	(101.26–103.72)	(320.42–323.08)
≥20.0	13.05 ± 0.03	39.26 ± 0.07	104.71 ± 1.13	318.51 ± 1.22
	(12.99–13.11)	(39.12–39.41)	(102.50–106.92)	(316.11–320.89)
* p* value	<0.001	<0.001	0.004	0.013
MetS (*n* = 2,296)				
25(OH)D (ng/ml) <10.0	13.25 ± 0.08	39.67 ± 0.10	97.02 ± 2.47	326.74 ± 3.17
	(13.10–13.40)	(39.28–40.07)	(92.17–101.87)	(320.52–332.96)
≥10.0, <20.0	13.31 ± 0.03	39.86 ± 0.07	98.25 ± 0.90	332.77 ± 1.15
	(13.26–13.36)	(39.72–40.00)	(96.50–100.01)	(320.51–325.02)
≥20.0	13.36 ± 0.04	39.97 ± 0.11	97.42 ± 1.40	322.92 ± 1.79
	(13.28–13.45)	(39.74–40.19)	(94.68–100.15)	(319.42–326.43)
*p* value	0.378	0.43	0.817	0.494

Non-MetS: Non-metabolic syndrome, MetS: Metabolic syndrome, *Adjusted for smoking, alcohol drinking, regular exercise, current menstruation, hormonal contraceptives, hormone-replacement therapy, SBP, DBP, BMI, WM, TC, TG, HDL-C, FBG, AST, ALT, and age.

## References

[1] Heeney MM, Andrews NC (2004). Iron homeostasis and inherited iron overload disorders: an overview. Hematol Oncol Clin North Am.

[2] Conrad ME, Umbreit JN, Moore EG (1999). Iron absorption and transport. Am J Med Sci.

[3] Cook JD, Flowers CH, Skikne BS (2003). The quantitative assessment of body iron. Blood.

[4] Rasheed H, Mahgoub D, Hegazy R (2013). Serum ferritin and vitamin D in female hair loss: do they play a role?. Skin Pharmacol Physiol.

[5] Lee JA, Hwang JS, Hwang IT, Kim DH, Seo JH, Lim JS (2015). Low vitamin D levels are associated with both iron deficiency and anemia in children and adolescents. Pediatr Hematol Oncol.

[6] Chon SJ, Choi YR, Roh YH (2014). Association between levels of serum ferritin and bone mineral density in Korean premenopausal and postmenopausal women: KNHANES 2008-2010. PLoS One.

[7] Williams MJ, Poulton R, Williams S (2002). Relationship of serum ferritin with cardiovascular risk factors and inflammation in young men and women. Atherosclerosis.

[8] Cho YS, Kang JH, Kim SA, Shim KW, Lee HS (2015). Association of serum ferritin and abdominal obesity and insulin resistance. Korean J Obes.

[9] Jehn M, Clark JM, Guallar E (2004). Serum ferritin and risk of the metabolic syndrome in U.S. adults. Diabetes Care.

[10] Holick MF (2007). Vitamin D deficiency. N Engl J Med.

[11] Ku YC, Liu ME, Ku CS, Liu TY, Lin SL (2013). Relationship between vitamin D deficiency and cardiovascular disease. World J Cardiol.

[12] Yoon H, Kim GS, Kim SG, Moon AE (2015). The relationship between metabolic syndrome and increase of metabolic syndrome score and serum vitamin D levels in Korean adults: 2012 Korean National Health and Nutrition Examination Survey. J Clin Biochem Nutr.

[13] Sim JJ, Lac PT, Liu IL (2010). Vitamin D deficiency and anemia: a cross-sectional study. Ann Hematol.

[14] Karadağ AS, Ertuğrul DT, Tutal E, Akin KO (2011). The role of anemia and vitamin D levels in acute and chronic telogen effluvium. Turk J Med Sci.

[15] Jeong DW, Lee HW, Cho YH (2014). Comparison of serum ferritin and vitamin D in association with the severity of nonalcoholic fatty liver disease in Korean adults. Endocrinol Metab (Seoul).

[16] Thomas CE, Guillet R, Queenan RA (2015). Vitamin D status is inversely associated with anemia and serum erythropoietin during pregnancy. Am J Clin Nutr.

[17] Kell DB, Pretorius E (2014). Serum ferritin is an important inflammatory disease marker, as it is mainly a leakage product from damaged cells. Metallomics.

[18] Nemeth E, Ganz T (2006). Regulation of iron metabolism by hepcidin. Annu Rev Nutr.

[19] Ilkovska B, Kotevska B, Trifunov G (2015). Elevated serum hepcidin and ferritin levels in patients with metabolic syndrome in macedonian population. IJAR.

[20] Martinelli N Traglia M Campostrini N Increased serum hepcidin levels in subjects with the metabolic syndrome: a population study PLoS One 2012 7 e48250. 23144745 10.1371/journal.pone.0048250 PMC3483177

[21] International Association for the Study of Obesity Task Force. (2000). The Asia-Pacific Perspective: Redefining Obesity and its Treatment.

[22] Yoon H, Jeon DJ, Park CE, You HS, Moon AE (2016). Relationship between homeostasis model assessment of insulin resistance and beta cell function and serum 25-hydroxyvitamin D in non-diabetic Korean adults. J Clin Biochem Nutr.

[23] Palacios C, Gonzalez L (2014). Is vitamin D deficiency a major global public health problem?. J Steroid Biochem Mol Biol.

[24] Hintzpeter B, Mensink GB, Thierfelder W, Müller MJ, Scheidt-Nave C (2008). Vitamin D status and health correlates among German adults. Eur J Clin Nutr.

[25] Zgaga L, Theodoratou E, Farrington SM (2011). Diet, environmental factors, and lifestyle underlie the high prevalence of vitamin D deficiency in healthy adults in Scotland, and supplementation reduces the proportion that are severely deficient. J Nutr.

[26] Pittas AG, Lau J, Hu FB, Dawson-Hughes B (2007). The role of vitamin D and calcium in type 2 diabetes. A systematic review and metaanalysis. J Clin Endocrinol Metab.

[27] Chiu KC, Chu A, Go VL, Saad MF (2004). Hypovitaminosis D is associated with insulin resistance and beta cell dysfunction. Am J Clin Nutr.

[28] Zughaier SM, Alvarez JA, Sloan JH, Konrad RJ, Tangpricha V (2014). The role of vitamin D in regulating the iron-hepcidin-ferroportin axis in monocytes. J Clin Transl Endocrinol.

[29] Bacchetta J, Zaritsky JJ, Sea JL (2014). Suppression of iron-regulatory hepcidin by vitamin D. J Am Soc Nephrol.

[30] Andıran N, Çelik N, Akça H, Doğan G (2012). Vitamin D deficiency in children and adolescents. J Clin Res Pediatr Endocrinol.

[31] Monlezun DJ, Camargo CA Jr, Mullen JT, Quraishi SA (2015). Vitamin D status and the risk of anemia in community-dwelling adults: results from the National Health and Nutrition Examination Survey 2001–2006. Medicine (Baltimore).

[32] Castro FD, Magalhães J, Carvalho PB, Moreina MJ, Mota P, Cotter J (2015). Lower levels of vitamin D correlate with clinical disease activity and quality of life in inflammatory bowel disease. Arq Gastroenterol.

[33] Suh YJ, Lee JE, Lee DH (2016). Prevalence and relationships of iron deficiency anemia with blood cadmium and vitamin D levels in Korean women. J Korean Med Sci.

[34] Hildrum B, Mykletun A, Hole T, Midthjell K, Dahl AA (2007). Age-specific prevalence of the metabolic syndrome defined by the International Diabetes Federation and the National Cholesterol Education Program: the Norwegian HUNT 2 study. BMC Public Health.

[35] Vieth R, Ladak Y, Walfish PG (2003). Age-related changes in the 25-hydroxyvitamin D versus parathyroid hormone relationship suggest a different reason why older adults require more vitamin D. J Clin Endocrinol Metab.

[36] Vanasse GJ, Berliner N (2010). Anemia in elderly patients: an emerging problem for the 21st century. Hematology Am Soc Hematol Educ Program.

[37] Zacharski LR, Chow B, Shamayeva G, Lavori P (2010). Effect of an interaction between age and ferritin level on clinical outcomes in peripheral arterial disease (PAD). Blood.

[38] Moreau R, Tshikudi Malu D, Dumais M (2012). Alterations in bone and erythropoiesis in hemolytic anemia: comparative study in bled, phenylhydrazine-treated and *Plasmodium*-infected mice. PLoS One.

[39] Talaei A, Mohamadi M, Adgi Z (2013). The effect of vitamin D on insulin resistance in patients with type 2 diabetes. Diabetol Metab Syndr.

[40] Garcia-Bailo B, El-Sohemy A, Haddad PS (2011). Vitamins D, C, and E in the prevention of type 2 diabetes mellitus: modulation of inflammation and oxidative stress. Biologics.

[41] Reichel H, Koeffler HP, Norman AW (1989). The role of the vitamin D endocrine system in health and disease. N Engl J Med.

[42] Reaven GM (1998). Banting lecture 1988. Role of insulin resistance in human disease. Diabetes.

[43] Esposito K, Giugliano D (2004). The metabolic syndrome and inflammation: association or causation?. Nutr Metab Cardiovasc Dis.

[44] Takeda E, Yamanaka-Okumura H, Taketani Y (2015). Effect of nutritional counseling and long term isomaltulose based liquid formula (MHN-01) intake on metabolic syndrome. J Clin Biochem Nutr.

[45] Smith EM Alvarez JA Kearns MD High-dose vitamin D3 reduces circulating hepcidin concentrations: a pilot, randomized, double-blind, placebo-controlled trial in healthy adults. Clin Nutr 2016 pii: S0261-5614(16)30148-0. 10.1016/j.clnu.2016.06.015. 10.1016/j.clnu.2016.06.015 PMC5191989 27402475

[46] Luo C, Wong J, Brown M, Hooper M, Molyneaux L, Yue DK (2009). Hypovitaminosis D in Chinese type 2 diabetes: lack of impact on clinical metabolic status and biomarkers of cellular inflammation. Diab Vasc Dis Res.

